# Supplementary figures and images for: Acute inflammation sensitizes knee-innervating sensory neurons and decreases mouse digging behavior in a TRPV1-dependent manner
Source: Neuropharmacology. 2018 Dec;143:49–62. doi: 10.1016/j.neuropharm.2018.09.014 (PMC6277850; doi:10.1016/j.neuropharm.2018.09.014)

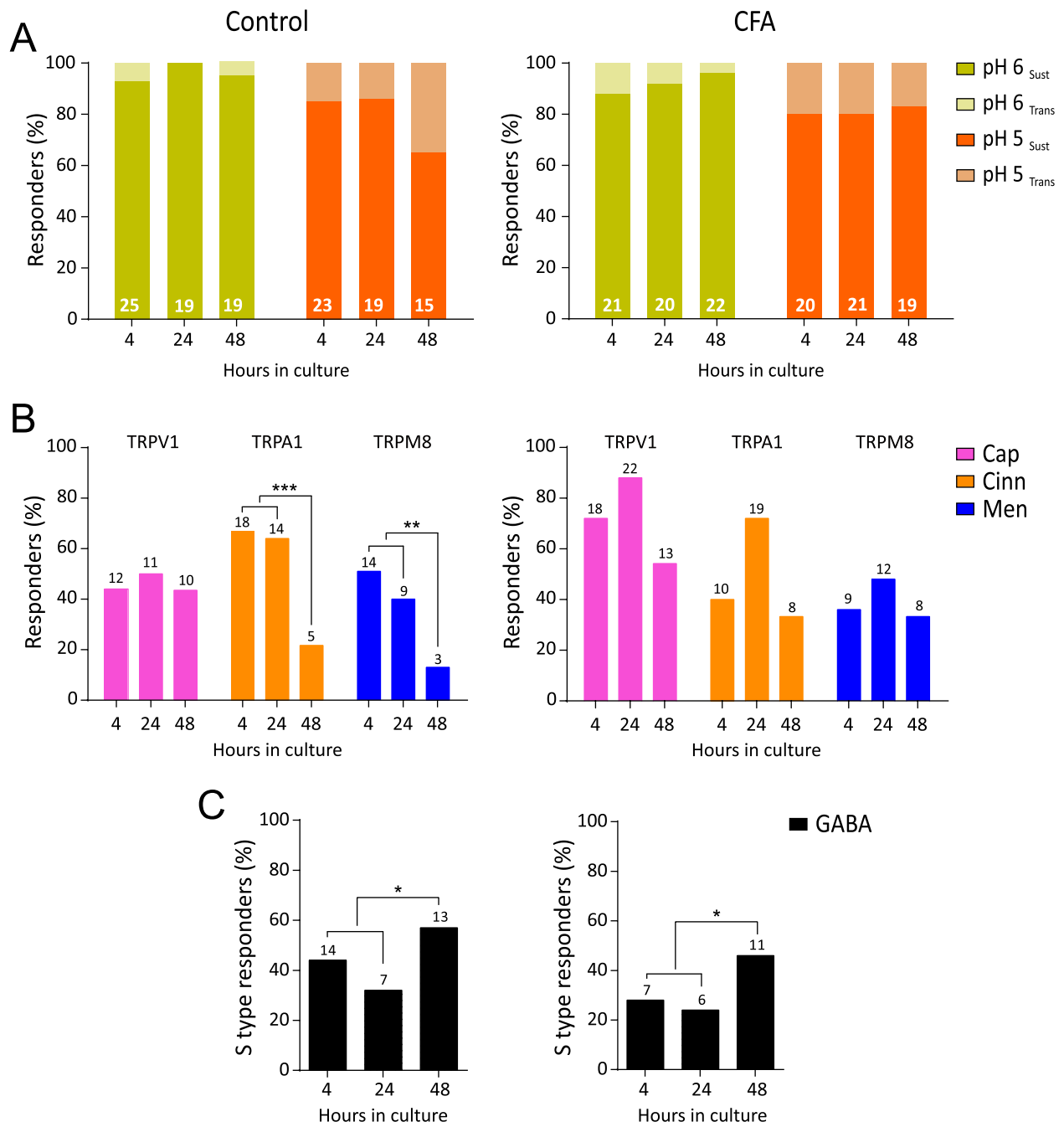

Supplement: Supplementary file 2 — Supplementary Digital Content 2Figure showing time-in-culture effects on acid, TRP agonist and GABA sensitivity of knee neurons after inflammation.pdf A) Proportion of Cntrl (left) and CFA (right) neurons responding to pH 6 (yellow) and pH 5 (orange) with a transient + sustained type current (light shade) and sustained only type current (dark shade) across 4, 24 and 48-h. B) Percentage frequency of Cntrl (left) and CFA (right) neurons sensitive to capsaicin (pink), cinnamaldehyde (orange) and menthol (blue) across 4, 24 and 48-h in culture. C) Percentage of Cntrl (left) and CFA (right) neurons that had a sustained GABA-evoked current across 4, 24 and 48-h in culture. * indicates p < 0.05, chi-sq test. The numbers above the bars indicate the number of responsive neurons. [file mmc2.pdf]
